# Total and Free Sugar Content of Canadian Prepackaged Foods and Beverages

**DOI:** 10.3390/nu8090582

**Published:** 2016-09-21

**Authors:** Jodi T. Bernstein, Alyssa Schermel, Christine M. Mills, Mary R. L’Abbé

**Affiliations:** 1 Department of Nutritional Sciences, Faculty of Medicine, University of Toronto, Toronto, ON M5S 3E2, Canada; jodi.bernstein@mail.utoronto.ca (J.T.B.); a.schermel@gmail.ca (A.S.); 2 Dalla Lana School of Public Health, University of Toronto, Toronto, ON M5T 3M7, Canada; chris.mills@mail.utoronto.ca

**Keywords:** sugars, free sugar, nutrition labelling, food composition, food supply, Canada, public health, policy

## Abstract

A number of recommendations for policy and program interventions to limit excess free sugar consumption have emerged, however there are a lack of data describing the amounts and types of sugar in foods. This study presents an assessment of sugar in Canadian prepackaged foods including: (a) the first systematic calculation of free sugar contents; (b) a comprehensive assessment of total sugar and free sugar levels; and (c) sweetener and free sugar ingredient use, using the University of Toronto’s Food Label Information Program (FLIP) database 2013 (*n* = 15,342). Food groups with the highest proportion of foods containing free sugar ingredients also had the highest median total sugar and free sugar contents (per 100 g/mL): desserts (94%, 15 g, and 12 g), sugars and sweets (91%, 50 g, and 50 g), and bakery products (83%, 16 g, and 14 g, proportion with free sugar ingredients, median total sugar and free sugar content in Canadian foods, respectively). Free sugar accounted for 64% of total sugar content. Eight of 17 food groups had ≥75% of the total sugar derived from free sugar. Free sugar contributed 20% of calories overall in prepackaged foods and beverages, with the highest at 70% in beverages. These data can be used to inform interventions aimed at limiting free sugar consumption.

## 1. Introduction

Excess consumption of free sugar (see Box 1 for definitions) has been associated with increased risk of obesity, cardiovascular disease, diabetes, and dental caries [1,2,3,4,5]. In fact, one study found that increased consumption of refined starches, like free sugar, are second only to trans fats in increasing risk of cardiovascular disease [6]. Thus, guidelines to limit intakes to a maximum of 5%–10% of calories/day [7,8,9,10,11] have emerged in many regions. Recommendations have also been made in Canada and other countries to decrease the affordability, availability, accessibility and exposure to products with excess free sugar [8,12,13]. Despite these calls to action, the lack of detailed data on the pervasiveness of sugar in the food environment [14] hinders the development of policies and programs to reduce free sugar consumption and associated health benefits with targeted interventions [15].

Box 1 **Definitions.**

- **“Free sugar”** is the sugar no longer in its naturally-occurring state (i.e., no longer in whole fruits, vegetables, unsweetened dairy, and grains) and can be consumed as is or incorporated into other foods [9]. Examples include table sugar, syrup, honey, fruit juice and nectars.
- **“Added sugar”** is the free sugar that has been added to foods [13], however regulatory definitions vary widely under different jurisdictions, some of which are currently under review [16].
- **“Naturally-occurring sugar”** is the sugar found naturally within whole foods (i.e., within whole fruits, vegetables, dairy, and some grains) [14].
- **“Total sugar”** is a combination of free sugar and naturally-occurring sugar and is currently the only type of sugar declared on the Nutrition Facts table (NFt) in Canada [17] and in many jurisdictions [16,18,19,20].
- **“Free Sugar Ingredients” (FSI)** are all mono- and disaccharides added to foods as well as those naturally-occurring in honey, fruit juices, and syrups (e.g., sugar, honey, maple syrup, molasses, fruit juice, glucose, fructose, agave, and corn syrup) [9].
- **“Sweeteners”** are food additives that are used to give products a sweet taste and can include sugar alcohols (e.g., malitol, xylitol, and sorbitol), non-nutritive sweeteners (e.g., aspartame, sucralose, and acesulfame-potassium), cyclamate sweeteners, or saccharin sweeteners [21] and are not considered FSI.

There are very limited data available on the free sugar contents of prepackaged foods and on consumption rates in Canada and globally [14]. This may be in part because free sugar is chemically indistinguishable from naturally-occurring sugar and as a result, contents must be calculated or supplied by food manufacturers. This has contributed to free sugar ingredients (FSI) being considered a “hidden” source of calories as it is not always obvious to consumers that they are present in food [22]. This phenomenon has been noted as a worry of Canadian parents [23]. Additionally, the various definitions used to describe sugar and inconsistencies in their components, make comparisons of food composition and sugar intakes problematic, increases the potential for confusion and misinterpretation and points to the need for uniform terminology [14]. Understanding the main sources and amounts of free sugar in Canadian foods will allow for monitoring trends in product formulations, reformulation efforts by the food industry, and Canadian intakes of free sugar overtime, that would otherwise be virtually impossible to measure.

Canadians consumed an average of 110 g (21.4% of calories) of total sugar per day in 2004 [24]. Although that report did not differentiate between total sugar and free sugar, another study used these total sugar intakes to estimate the average added sugar consumption of Canadians at 11%–13% of calories [24,25]. These authors estimated the proportion of total sugar coming from added sugar by assuming each of the top food categories contributed either naturally-occurring sugar or added sugar [25]. These total and added sugar consumption rates were based on food composition information obtained from the Canadian Nutrient File (CNF) database, the national nutrition database maintained by Health Canada [26,27]. However, using the CNF to assess sugar in the food supply poses several challenges, including its lack of scheduled, systematic and comprehensive updating, and its lack of brand-specific data [27]. Such data are required for analyzing a rapidly changing food supply, which can vary widely in free sugar content and the use of sweeteners. In contrast with these earlier assessments, more precise estimates of total, added, and free sugar intakes are needed to inform and evaluate relevant public health initiatives.

To obtain a more accurate assessment of the types and amounts of sugar in the Canadian food supply, data need to be reconciled using comprehensive, current, and accurate food composition data [14] along with systematic calculations of free sugar content. Acknowledging this need, in 2014 the Heart and Stroke Foundation of Canada (HSFC) called upon researchers to quantify the amount of free sugar in the Canadian food supply [8]. The overall purpose of this study is to provide a detailed and systematic evaluation of free sugar contents in a large representative sample of Canadian prepackaged foods that can serve as a benchmark to support and measure public health interventions and monitor free sugar consumption. Specific objectives include: (1) determining the amount of free sugar in Canadian prepackaged foods using a step-by-step decision algorithm tailored for use on a large, systematically collected, branded food composition database; (2) assessing total sugar and free sugar contents by food group and by detailed subcategory; and (3) conducting the first comprehensive assessment of the use of free sugar ingredients (FSI) and sweeteners in prepackaged foods and beverages. 

## 2. Materials and Methods

### 2.1. Food Label Information Program (FLIP) Database

The Food Label Information Program (FLIP) is a database of Canadian food and beverage package labels by brand name that is updated every three years at the University of Toronto (U of T). The purpose of the FLIP is to provide detailed assessments of the nutrition information found on the labels of food products in the Canadian marketplace, and to monitor changes over time. To date, two phases of the FLIP have been completed. The first phase, with data acquired in 2010/2011 (FLIP 2010), is described elsewhere [28]. The second phase, FLIP 2013, is described in this paper. The FLIP 2013 contains nutrition information for 15,342 unique products. Data collection took a similar approach as the FLIP 2010 with regards to acquiring food information from the top selling grocery retailers, although it was fully digitalized to enhance the ease and efficiency of collection and analysis. Food composition database software (University of Toronto and Dietitians of Canada, Toronto, ON, Canada) (web and mobile) was developed for FLIP 2013 in collaboration with the Dietitians of Canada, resulting in a shorter and more efficient food collection and data processing approach. 

#### 2.1.1. FLIP 2013 Data Collection

Data acquisition occurred between May and September 2013, and was carried out in the Greater Toronto Area and Ottawa, Ontario, and Calgary, Alberta. Data were collected from major outlets of the four largest grocery chains in Canada (Loblaws, Metro, Sobeys, and Safeway), representing 75.4% of the grocery retail market share [29]. A Smartphone application was developed and used to scan and store the Universal Product Code (UPC), and to photograph all sides of food and beverage packages, and capture price. By systematically scanning the grocery store shelves, every food product with a Nutrition Facts table (NFt), including all available national and private label brands were collected. Seasonal products (e.g., eggnog, Easter chocolates), Natural Health Products (e.g., supplements), baby/toddler foods, and products that did not have a Canadian NFt (e.g., unpackaged fruits, vegetables) were excluded from the data collection. Food products sold at multiple retailers (such as national brand products) were captured only once. When multiple sizes of a product were available, only one size was sampled, but all flavours and varieties of a product were collected. Information collected for each product included the UPC, company, brand, price, NFt information, ingredients, container size, nutrient content claims, disease risk reduction claims, function claims, front of pack symbols, children’s marketing, other claims (e.g., organic, natural, and gluten-free), and date and location of sampling. 

#### 2.1.2. FLIP 2013 Data Processing

Upon scanning the UPC code, foods that had not already been collected in this phase were automatically assigned a product ID and photos uploaded onto the FLIP website for data processing. The FLIP website allowed for efficient data entry using dropdown menus (e.g., to assign foods to specific categories or to indicate the presence of different nutrition claims), and used Optical Character Recognition technology to automatically extract data from the NFt and ingredients list. The FLIP database, run on a Microsoft SQL server, also enabled users to generate data outputs and reports in Microsoft Excel for further statistical analyses.

Food products were classified under multiple categorization systems. Categorization systems used included Schedule M of the Food and Drug Regulations (B.01.001) [30], as well as Health Canada’s sodium categories for guiding benchmark sodium levels [31]. These classification systems were also used to create similar systems specific to other nutrients, e.g., trans-fat and the sugar focused food categories used for the present study.

When required, data for some food products were also calculated for the “as consumed” form (e.g., cake mixes, drink powders, and condensed soups) using ESHA Food Processor software and food composition data from the Canadian Nutrient File [32] in order to be comparable to the prepared versions within that particular food category. In addition, for some products, serving grams were converted to millilitres and vice versa for consistency across all products within a food category. The database underwent extensive quality control checks including verification of inputted nutrient contents using Atwater factors and outliers to check for erroneous values, and multiple reviews for NFt, Ingredient Lists, gram to millilitre conversions, and food group categorizations. Excluded from this analysis were meal replacement beverages, which are indicated for special dietary use (*n* = 55), and products with missing total sugar declarations (*n* = 28) for a total of 15,259 products in the present study.

#### 2.1.3. FLIP 2013 Sugar-Focused Food Categories

Products were classified into 17 sugar-focused major food groups, including 77 major subcategories, and 207 minor categories. Sugar-focused categories were created based on Schedule M food categories as outlined in the Canadian Food and Drug Regulations [30], as well as Health Canada’s sodium-focused categories [33]. These categories were further divided or combined on the basis of sugar and sweetener ingredients, intended use, and food type to ensure categories containing like products. 

### 2.2. Assessment of Free and Total Sugar Content and Use of Free Sugar Ingredients and Sweeteners

Free sugar is chemically indistinguishable from naturally-occurring sugar [34]. As there is no declaration of free sugar content on the NFt, an algorithm was developed to derive free sugar contents which was guided by a published, systematic methodology for estimating added sugars [35,36]. The U of T free sugar algorithm steps, to be conducted in sequential order, as well as the proportion of free sugar contents calculated at each step, are outlined in Table 1. For the purpose of this analysis, free sugar ingredients (FSI) refers to any free sugar ingredient that meets the WHO definition for free sugar including sugar, syrup, honey, fruit juices, and other sweetening agents [9]. “Sweeteners”, as defined by the Canadian Food Inspection Agency as a food additive that is used to give products a sweet taste and can include sugar alcohols (e.g., malitol, xylitol, and sorbitol), non-nutritive sweeteners (e.g., aspartame, sucralose, and acesulfame-potassium), cyclamate sweeteners, or saccharin sweeteners [21] were not considered FSI. Presence of FSI and sweeteners were identified by searching the Ingredient List of each product and the ingredients required in product preparation as stated on the package. The means and distributions of total sugar content, obtained from the NFt, and of the calculated free sugar content were reported as g per 100 g or g per 100 mL (the latter for beverages and desserts), by food group, subcategory, and minor category. Free sugar content was calculated as a percent of total sugar and as a percent of energy, the latter to allow for comparisons with maximum intake guidelines, which are usually presented as a percent of calories. All calculations were conducted on the sugar content of the “as consumed” version of the product.

### 2.3. Statistical Analysis

Mean, SD, and quartiles (min, 25th, 50th, 75th, max) were determined for total sugar and free sugar content. The percent of total sugar and of calories derived from free sugar were presented as proportions. Categorical variables (e.g., presence of FSI and sweetener ingredients) were presented as frequencies (percentages). All statistical analyses were conducted using SAS version 9.4 (SAS Institute Inc., Cary, NC, USA).

## 3. Results

### 3.1. Use of Free Sugar Ingredients and Sweeteners

**Figure 1:**
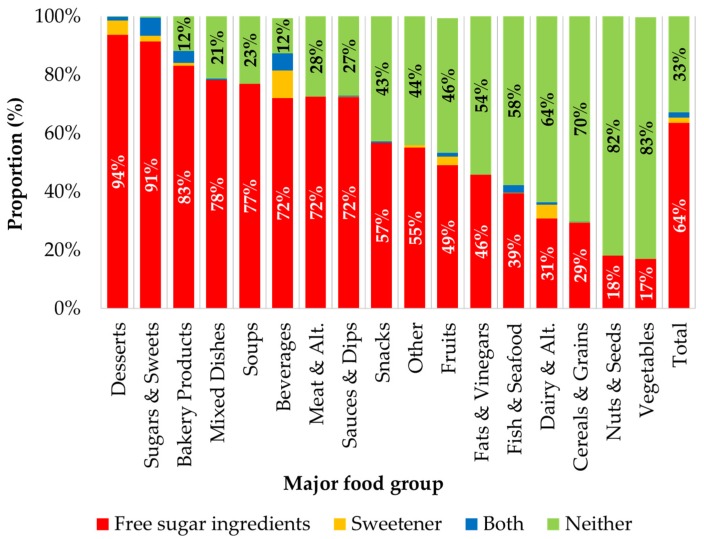
Proportion (%) of prepackaged foods and beverages containing free sugar ingredients, sweeteners, a combination of both, or neither, by major food category and overall (*n* = 15,259). Proportions labelled on the figure only when value is >10%. “FSI” are those defined in Table 2. ”Sweeteners” refers to all non- or low-caloric sweetening agents as defined by the Canadian Food Inspection Agency, including sugar alcohols (e.g., xylitol, and sorbitol), and non-caloric or artificial sweeteners (e.g., sucralose, and aspartame) [20]. Abbreviations: Alt. = Alternatives.

**Figure 2:**
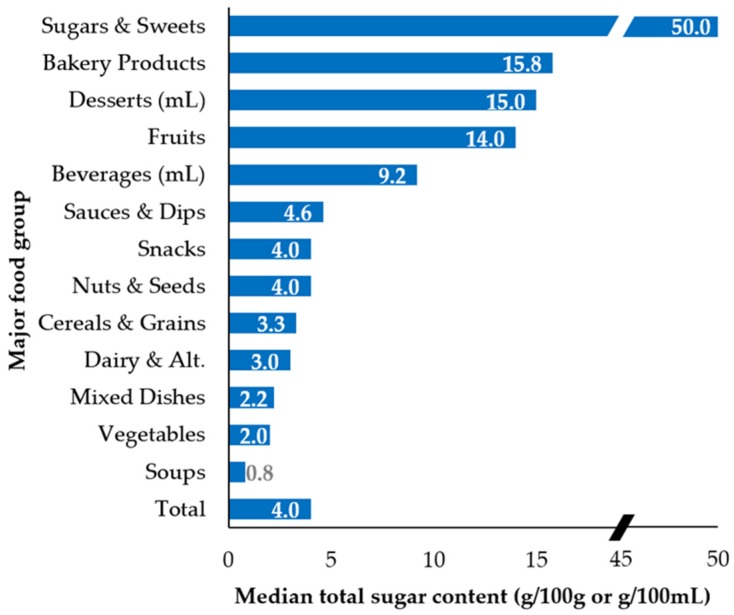

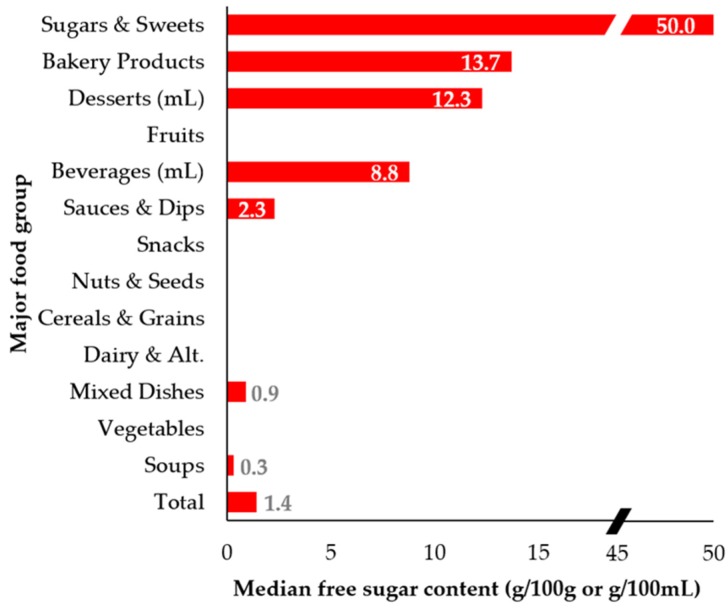
Median total sugar and free sugar content (g/100 g or g/100 mL) by major food group and overall (*n* = 15,259): (**Top**) median total sugar content; and (**Bottom**) median free sugar content. Categories with 0 g/100 g or 100 mL median total sugar and free sugar (i.e., other foods and beverages; fats, oils, and vinegars; meat and alternatives; and fish and seafood) are not shown. (▰) denotes a break in the x-axis between 20 and 45 g/100 g.

Overall, 63.5% of prepackaged foods contained a FSI, 1.9% contained a sweetener, another 1.8% contained both a FSI and a sweetener, and the remaining 32.9% contained neither (Figure 1). There were 152 unique FSI found in this representative sample of Canadian prepackaged foods, not including variations in spelling, indicators of quality, purity, or origin (e.g., organic maple syrup, 100% pure agave, and Canadian honey) or specific flavours of fruit juice (e.g., apple juice, and grape juice concentrate) (Table 2). The most common types of FSI found in Canadian food and beverage products were sugar (dried or granulated) identified in 49.3% of products, glucose in 19.3%, and corn syrup in 10.7% (Table 2). Major food categories with the highest proportion of products containing FSI were desserts (93.6%), sugars and sweets (91.4%), and bakery products (83.1%) (Figure 1). These were also the most total sugar and free sugar dense food groups (Figure 2).

### 3.2. Median Total and Free Sugar Content

Median free sugar content overall was 1.4 g per 100 g (or 100 mL), about one-third of the median total sugar content (4.0 g per 100 g/mL) (Figure 2). Fruits had the fourth highest median total sugar content (14.0 g/100 g) but was among the lowest free sugar containing food groups with 0 g/100 g. This was followed by beverages with a median 9.2 g/100 mL total sugar and 8.8 g/100 mL free sugar. All other food groups contained about half or less than these total and free sugar levels. For free sugar, this drop was even more dramatic, with all other categories containing less than 2.5 g/100 g. When examining the food supply in detail by subcategories (Table 3), the top total sugar containing subcategories were sugar (100 g/100 g), fruit snacks (72 g/100 g), dried fruits (55 g/100 g), dessert toppings and fillings (53 g/100 mL), confectionery (51 g/100 g), and sweet condiments (50 g/100 g). The top free sugar containing subcategories were also sugar (100 g/100 g), dessert toppings and fillings (53 g/100 mL), confectionery (51 g/100 g), and sweet condiments (50 g/100 g), however, dried fruits and fruit snacks were not among the top free sugar subcategories with 0 g/100 g median free sugar content. 

### 3.3. Free Sugar as a Percent of Total Sugar

**Figure 3:**
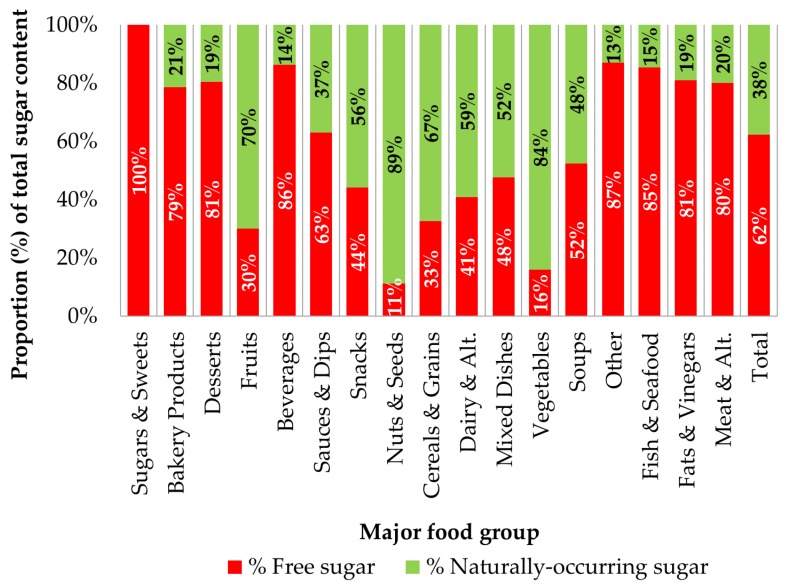
Free sugar and naturally-occurring sugar as a proportion (%) of total sugar by major food group and overall (*n* = 15,259). Free sugar and naturally-occurring sugar as a percent of total sugar was calculated for each product and the average of those results is presented here.

Overall, free sugar accounted for 62% of the total sugar in prepackaged foods and beverages; the remainder was from naturally-occurring sources of sugar (Figure 3). In nearly half of the major food categories examined, free sugar contributed at least 75% of total sugar. This ranged from 100% of the total sugar in the food group sugars and sweets, to 11% of the total sugar in the nuts and seeds food group. Of the top sugar-dense food groups, free sugar as a proportion of total sugar for bakery products was 79%, for desserts 81%, and for beverages 86%. Because some food categories contain very little total sugar, the addition of small amounts of free sugar can result in the percentages appearing quite high. For example, free sugar as a proportion of total sugar in fats and vinegars (81%), other foods and beverages (87%), and fish and seafood (85%) are high, but all had a median free sugar content of 0 g/100 g. A more detailed evaluation of free sugar at the subcategory and minor category level (Table 3), revealed that free sugar accounted for 100% of the total sugar in cookies, energy drinks, fruit drinks, soft drinks, sports drinks, dessert toppings and fillings, mayonnaise, bacon, eggs, and all subcategories of the sugars and sweets food group. Additionally, free sugar accounted for >90% of the total sugar in many baked desserts, muffins, cakes, cereal and granola bars, ready-to-eat cereals, and several minor categories of condiments and sauces. 

### 3.4. Contribution of Free Sugar to Total Calories

**Figure 4:**
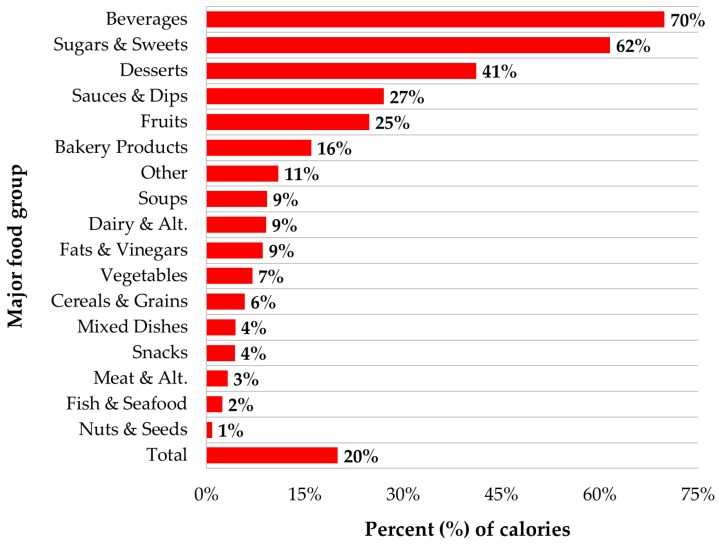
Free sugar as a percent of calories, by major food group and overall (*n* = 15,259).

Free sugar contributed on average 20% of calories in the prepackaged foods and beverages evaluated (Figure 4), with content ≥10% of calories in seven of the 17 major food groups, including beverages (70%), sugars and sweets (62%), and desserts (41%).

## 4. Discussion

With a number of recommendations to enact policies and initiate programs that support limiting sugar intakes, it is imperative that baseline information on the types and amounts of sugar in Canadian foods and beverages be available for researchers, policy-makers, healthcare practitioners and consumers to make evidence-based decisions. This study was conducted to meet this need and is the first to systematically calculate free sugar content and to report on the total and free sugar contents and the use of FSI and sweeteners in a large representative sample of Canadian prepackaged foods and beverages. 

Alarmingly, free sugar in products contributed an average of 20% of calories in prepackaged foods and beverages, which is in excess of WHO free su34gar and US Dietary Guidelines added sugar intake recommendations at a maximum of 10% of calories [9,10]. Consumption of products with excessive free sugar contents, enhances the likelihood of exceeding these recommendations [38]. Some of the more sugar-dense food groups identified in this study, foods such as sweet bakery products, frozen desserts, confectionery, and sugar-sweetened beverages, are not recommended in Eating Well with Canada’s Food Guide [39]; yet these “other foods” contributed more than one-third (34.7%) of the total sugar Canadians consumed in 2004 [24,39]. 

This study identified 152 different names for FSI used in Canadian Ingredient Lists, highlighting the challenge faced by consumers trying to limit their intakes of free sugar. These FSI were ubiquitously found throughout the food supply and were present in every major food group. Data on FSI use in Canadian foods (65.4%), are slightly lower than that reported in the US where 74% of packaged foods were reported to contain added sugar ingredients in 2005–2009 [40]. 

Results of this study also identified that sweeteners were used in less than 5% of products. Not surprisingly, they are most often used in food groups with the highest total and free sugar contents. Efforts to reduce added or free sugar intakes have raised concerns that reformulation will not result in a reduction in calories (e.g., due to an increase in refined starches, fats) [41], or will increase the use of artificial sweeteners [42,43]. The evidence of the health effects or benefits of sweetener use in the long-term is inconclusive [42,43]. Thus, some recommendations to reduce sugar content of prepackaged foods stipulate that this should not be met with the subsequent addition of sweeteners [8,22]. 

The data presented here can be useful to support several interventions aimed at reducing intakes of free sugar. Firstly, reformulation of existing products and the development of new products to be lower in sugar have been suggested as ways to decrease the health burden associated with excess free sugar consumption [44]. This strategy, similar to the sodium reduction strategies in Canada [31] and other countries could likely be repeated for free sugar [22]. The data provided here would support such a strategy and shows that there is a wide range of free sugar content within a food category, demonstrating that products with lower free sugar contents are achievable, feasible, palatable, and sellable as shown in Table 3. This type of intra-category assessment of distributions can be used to develop the benchmarks needed for concerted efforts at free sugar reduction. Secondly, data on the free sugar content of prepackaged foods can be linked to national dietary intake surveys to provide the first evaluation of Canadian free sugar consumption. Subsequently, consumption data can be used to predict and monitor health outcomes associated with varying levels of free sugar intakes. Thirdly, the data on sweetener use and FSI use provided here can act as a baseline by which to compare future trends. Additionally, this data can be used to support consumer educational efforts to emphasize the many names for FSI, thereby helping consumers to more easily identify products that contain free sugar as well as the main food sources. One novel feature of the recently proposed Canadian nutrition labelling changes to address this concern is the proposal to group all sugar based ingredients in brackets after the word “Sugars” and be placed in the ingredient list in descending order according to the combined weight rather than scattered throughout the Ingredient List [45].

Limitations of this study include the use of declared sugar contents from the NFt, rather than laboratory analyses. NFt declarations are subject to the Canadian Food Inspection Agency’s rounding rules and can vary up to 20% from the actual analyzed value [46]. Given the vastness of the database, analysis of each product was not a feasible option. However, a study evaluating the accuracy of the declared nutrient contents of 1000 Canadian foods found only 13% of foods with unsatisfactory values (>20% difference from analyzed) for sugar contents [47]. Additionally, there are no chemical analyses available to differentiate free or added sugar from total sugar content. To account for this, the calculation of free sugar contents was based on a similar algorithm developed by Louie and colleagues [36], to estimate added sugar contents in the Australian food supply, that has been shown to have high levels of inter-researcher repeatability [36]. The most subjective step in the U of T free sugar algorithm, Step 5, where substitute added sugar values are chosen, was done by two people and consensus was reached for any discrepancies; however, this step was only required for 2.6% of foods. Finally, the FLIP 2013 database did not include all prepackaged foods and beverages available in Canada, but rather a systematically collected and large representative subset, comprising over three-quarters of the Canadian grocery retail market share.

## 5. Conclusions

In summary, this is the first study in Canada to calculate free sugar contents and these data provide the first detailed overview of FSI and sweetener use, and of total and free sugar contents of Canadian prepackaged foods and beverages. Using the detailed free sugar algorithm and the information from the NFt and Ingredient List, free sugar content was calculated for 96.5% of the foods and only imputed for 3.5%. The method described here can be employed for use on other large branded food databases. Findings can be used to inform, monitor, and evaluate interventions to limit excess sugar consumption, and indicate areas of concern for reformulation or educational efforts. The extensive data provided in this study can be incorporated into food composition databases and can be used to measure free sugar intakes with national nutrition surveys where it is currently not available [24], and determine intakes, particularly for vulnerable groups such as children and adolescents [8,25], compared to recommendations from the WHO [9].

## Figures and Tables

**Table 1:** Step-by-step method for calculating free sugar content of foods and beverages in the University of Toronto’s Food Label Information Program (FLIP) database 2013 and number of foods at each step (total *n* = 15,259).

Description	*n* (%) ^1^
Step 1: Products that contain 0 g total sugar as declared on the NFt. Free sugar value = 0 g/100 g.	3586 (23.5%)
Step 2: Products that contain no FSI ^2^ listed in the Ingredient List. Free sugar value = 0 g/100 g.	2620 (17.2%)
Step 3: Products that contain ingredients which contribute no, or a minimal amount of naturally-occurring sugars (i.e., fruits, vegetables, dairy, grains). Free sugar value = 100% of the declared total sugar content (e.g., soft drinks, fruit drinks) ^3^.	1642 (10.8%)
Step 4: Products that contain both naturally-occurring sugars and FSI, were compared to similar products without FSI (from steps 1 and 2) from the same subcategory (i.e., RTE breakfast cereals with FSI vs. RTE breakfast cereals without FSI) or minor category (i.e., milk, flavoured vs. milk, plain). The following equation [36] was used to calculate free sugar contents: $\frac{100\;\times \;\left(\mathrm{Sugar}\;\mathrm{per}\;100\;g\;\mathrm{unsweetened}-\mathrm{Sugar}\;\mathrm{per}\;100\;g\;\mathrm{sweetened}\right)}{\left(\mathrm{Sugar}\;\mathrm{per}\;100\;g\;\mathrm{unsweetened}-100\right)}$ When possible, specific comparisons were made based on main ingredients, flavours, specific nutrient contents, or product formats (i.e., fat-free sweetened yogurt vs. fat-free plain yogurt). Calculations resulting in negative free sugar contents (<0 g) were rounded up to 0 g.	6876 (45.1%)
Step 5: Products that do not have unsweetened comparators in the same subcategory in FLIP, were assigned a free sugar value based on a substitute value from the USDA Database for the Added Sugars Content of Selected Foods [37]. A free sugar value that matches the proportion of total sugar from added sugar in a product from the USDA database was assigned. For example, a product was assigned a free sugar value at 80% of total sugar if the comparable USDA database product had 80% of the total sugar coming from added sugars.	402 (2.6%)
Step 6: Products with no comparator in Step 5, were assigned a value reflective of the proportion of total sugar coming from free sugar in products within the same food group (e.g., a chocolate cake is assigned a free sugar value based on the percent of total sugar derived from free sugar content of other products in bakery products). Only products that contained free sugar (steps 3–5) were included in the calculation.	133 (0.9%)

^1^ Numbers presented represent the number and proportion (%) of products calculated at each step. All calculations were done for products in the “as consumed” form. For determination of free sugar contents in the “as consumed” form: total sugar content of the “as consumed” form was used in place of the total sugar content in the “as purchased” form as declared on the NFt; FSI added according to the package directions were treated the same as a FSI in the Ingredient List (Step #2–6); “as consumed” versions of substitute products were used when available (i.e., brownies instead of brownie mix) (Step #5); ^2^ FSI (free sugar ingredients) for this study refers to all mono- and disaccharides added to foods as well as those naturally-occurring in honey, fruit juices, and syrups (e.g., sugar, honey, maple syrup, molasses, fruit juice, glucose, fructose, agave, and corn syrup) [9]; ^3^ All products in the energy drinks, fruit drinks, soft drinks, and sports drinks categories that reached Step 3 were considered to contain a minimal amount of naturally-occurring sugars. Abbreviations: FLIP = Food Label Information Program; NFt = Nutrition Facts table; FSI = free sugar ingredients; RTE = Ready-to-eat; USDA = United States Department of Agriculture.

**Table 2:** Types of free sugar ingredients (FSI) identified in the FLIP 2013 database of Canadian prepackaged foods and beverages, by descending order of use (*n* = 15,259).

Type	Examples ^1^	*n* (% Foods with FSI) ^2^
Sugar (sucrose), dried and granulated	sugar, sucrose, brown sugar, cane sugar, pure sugar cane, pure cane sugar, raw cane sugar, powdered sugar, golden sugar, golden cane sugar, granulated cane sugar, granulated sugar cane juice, beet sugar, refined cane sugar, icing sugar, dried sugar cane juice, demerara sugar, light brown sugar, refinery syrup powder, invert sugar, evaporated cane juice, evaporated cane juice crystals, evaporated milled sugar, milled cane sugar, evaporated sugar cane juice, caster sugar, coarse sugar, turbinado sugar, natural cane sugar, turbinado cane sugar, white sugar, whole cane sugar, yellow sugar, dehydrated cane juice, dehydrated cane sugar, natural evaporated cane juice, confectioner’s sugar, fondant sugar, raw sugar, evaporated cane sugar, dehydrated cane syrup, dark brown sugar	7517 (49.3%)
Glucose	glucose, glucose solids, glucose syrup, dextrose, dextrose anhydrous, dextrose syrup, anhydrous dextrose, dried glucose syrup, dextrin syrup	2939 (19.3%)
Corn syrup	corn syrup, corn syrup powder, corn syrup solids, high maltose corn syrup, dried corn syrup extract, glucose-fructose, caramelized glucose-fructose, corn malt syrup, fructose- glucose, glucose-fructose syrup, corn sweetener	1626 (10.7%)
Fruit juice	concentrated fruit juice, fruit juice, fruit juice concentrates, fruit juice from concentrate	1202 (7.9%)
High-fructose corn syrup ^3^	high fructose corn syrup, sugar/glucose-fructose, sugar/fructose-glucose, sugar and/or glucose-fructose, sugar and/or fructose-glucose	873 (5.7%)
Molasses	molasses, dehydrated molasses, powdered refiner’s molasses, black molasses, blackstrap molasses, dried molasses, refiner’s molasses, cane juice molasses, dry blackstrap molasses, dry molasses, fancy molasses, fancy molasses powder, cooking molasses, molasses granules, molasses powder, molasses solids	706 (4.6%)
Honey	honey, liquid honey, amber honey, pasteurized honey, honey granules, honey powder, honey solids, creamed honey, dried honey, granulated honey, raw honey, buckwheat honey, dried honey powder, dry honey, white honey	625 (4.1%)
Sugar (sucrose) syrups ^4^	cane sugar syrup, sucrose syrup, dried cane syrup, cane syrup, cane refiner’s syrup, refined sugar syrup, burnt sugar syrup, invert cane syrup, golden syrup, refiner’s syrup, invert sugar syrup, caramel, caramel sugar syrup, caramel syrup, evaporated cane syrup, liquid invert sugar, liquid sugar, liquid sucrose, evaporated cane juice syrup, sugar cane syrup, treacle	514 (3.4%)
Other syrups	brown rice syrup, apple cider syrup, apple syrup, rice syrup, malt syrup, barley malt syrup, malted barley syrup, tapioca syrup, raisin syrup, sorghum syrup, wheat syrup	439 (2.9%)
Fructose	fructose, fructose solids, fructose syrup, crystalline fructose	324 (2.1%)
Other sugars	potato syrup solids, palm sugar, tapioca sugar, tapioca syrup solids, lactose, coconut sugar, oat syrup solids, maltose, isomaltose	272 (1.8%)
Maple syrup	maple syrup, dehydrated maple syrup, maple sugar	72 (0.5%)
Agave	agave, agave nectar	27 (0.2%)

^1^ 152 unique FSI were identified, not including different spellings, “organic” variations of nomenclature (e.g., organic cane sugar), claims of origin (e.g., Canadian maple syrup), claims of purity (e.g., 100% pure agave) and specific flavours of fruit juice (e.g., apple juice, pear juice), are presented in descending order of use; determined from the number of products that contained each FSI; ^2^ Combined percentage of foods containing a FSI exceeds 100% because 4642 (30.4%) of the food supply or 46.6% of the products with a FSI contained more than 1 type of FSI; ^3^ Labelling terminology used in Canada for high-fructose corn syrup; ^4^ Caramel used for colour, when indicated within the ingredient list, was not considered a FSI. Abbreviations: FLIP = Food Label Information Program.

**Table 3:** Total and free sugar contents (g per 100 g or 100 mL) and average free sugar as a proportion of total sugar (%) in FLIP 2013 by food group, subcategory, and minor category (*n* = 15,259) ^1^.

Food Group, Subcategory, and Minor Category	*n*	Total Sugar (g/100 g or 100 mL)						Free Sugar (g/100 g or 100 mL)						Free Sugar as a Percent of Total Sugar ^2^
		$\bar{X}$ (SD)	Min	25th	50th	75th	Max	$\bar{X}$ (SD)	Min	25th	50th	75th	Max	
Bakery Products	2197	17 (15)	0	4	16	29	94	16 (14)	0	2	14	28	94	79%
*Baked Breakfast*	*123*	*10 (7)*	*1*	*5*	*7*	*10*	*38*	*5 (8)*	*0*	*0*	*2*	*5*	*34*	*29%*
Croissants	6	11 (5)	6	7	8	17	18	5 (6)	1	1	2	12	12	38%
Pancakes, Waffles, French Toast	92	7 (3)	1	5	6	9	24	2 (3)	0	0	0	3	20	18%
Tea Biscuits and Scones	14	12 (8)	4	7	11	14	28	9 (8)	0	3	7	11	25	59%
Toaster Pastries	11	26 (9)	15	17	24	36	38	22 (10)	10	12	19	32	34	80%
*Baked Desserts*	*88*	*30 (11)*	*8*	*22*	*28*	*39*	*50*	*27 (10)*	*6*	*18*	*24*	*36*	*46*	*88%*
Brownies/Squares	39	39 (6)	22	35	40	43	50	36 (7)	18	33	36	40	46	92%
Doughnut, Cake	12	23 (6)	12	19	23	27	30	21 (5)	11	17	20	24	29	92%
Doughnut, Yeast	5	19 (4)	15	17	17	20	24	17 (3)	14	16	16	18	23	93%
Fruit-Filled Pastries	8	24 (4)	19	20	24	28	31	17 (3)	13	14	17	19	21	69%
Other Pastries (e.g., eclairs)	14	23 (11)	8	14	21	27	50	19 (10)	6	11	18	20	41	80%
Sweet Buns (e.g., cinnamon rolls)	10	23 (6)	11	22	25	27	32	21 (6)	9	21	23	25	29	91%
*Bread Products*	*548*	*6 (7)*	*0*	*2*	*3*	*6*	*36*	*4 (7)*	*0*	*0*	*2*	*4*	*35*	*58%*
Bagels	37	5 (3)	2	4	5	7	16	3 (3)	0	1	3	5	15	62%
Bread w/ Additions (e.g., garlic bread)	31	2 (3)	0	0	2	3	14	1 (1)	0	0	0	1	4	27%
Bread w/ Raisins	15	16 (6)	5	9	16	18	24	12 (8)	0	4	14	17	23	72%
Diet Bread	6	4 (1)	2	2	4	5	5	2 (1)	1	1	2	3	3	52%
English Muffins	24	2 (2)	0	2	2	2	13	0 (1)	0	0	0	0	2	19%
Flatbreads (e.g., pita, naan, tortillas)	131	3 (4)	0	0	2	4	20	2 (4)	0	0	1	3	19	60%
Hearth Bread	69	2 (2)	0	0	2	2	9	1 (2)	0	0	0	1	9	39%
Muffins and Quick Breads	57	24 (7)	7	20	25	28	36	22 (7)	0	19	23	27	35	93%
Pantry Bread and Rolls	178	4 (2)	0	3	4	5	20	2 (2)	0	1	2	3	8	56%
*Cake*	*246*	*30 (9)*	*13*	*23*	*28*	*36*	*56*	*27 (8)*	*10*	*20*	*25*	*32*	*52*	*89%*
Cake Mixes	52	23 (5)	16	20	22	24	36	22 (5)	15	19	20	23	36	95%
Cakes w/Icing/Filling	21	32 (7)	13	29	34	36	38	27 (6)	10	24	28	31	36	86%
Cheesecakes	35	25 (4)	18	22	25	27	32	21 (3)	15	18	21	23	27	83%
Coffee Cakes w/o Icing/Filling	42	28 (5)	19	25	27	30	43	26 (4)	18	23	26	28	41	95%
Cream, Custard and Mousse Cake	13	22 (4)	16	18	21	23	32	18 (4)	13	15	18	19	26	83%
Cupcakes	28	43 (6)	29	40	42	46	56	37 (7)	24	33	36	41	51	85%
Ice Cream Cakes	11	26 (5)	13	23	26	28	35	21 (5)	11	19	22	23	29	83%
Sauce Cakes	4	27 (2)	25	25	26	28	29	22 (1)	20	21	22	23	24	83%
Snack Cakes	29	41 (8)	24	36	43	46	54	37 (8)	23	31	37	44	52	91%
Sponge Cakes w/o Icing/Filling	6	36 (4)	30	34	37	38	43	36 (4)	30	33	37	37	42	99%
Upside-down and Fruit Cakes	5	31 (2)	28	30	31	32	34	26 (2)	23	25	26	27	28	83%
*Cereal/Granola Bars*	*202*	*30 (7)*	*11*	*24*	*31*	*35*	*57*	*27 (7)*	*11*	*22*	*27*	*32*	*53*	*91%*
w/ Filling or Coating	101	33 (6)	18	29	34	37	45	29 (6)	17	24	29	34	41	88%
w/o Filling or Coating	101	27 (7)	11	23	27	32	57	26 (7)	11	21	25	30	53	94%
*Cookies*	*412*	*32 (10)*	*0*	*27*	*32*	*38*	*94*	*32 (10)*	*0*	*27*	*32*	*38*	*94*	*100%*
Chocolate Chip	81	32 (7)	0	30	33	36	43	32 (7)	0	30	33	36	43	100%
Chocolate Covered	39	35 (11)	0	29	37	41	56	35 (11)	0	29	37	41	56	100%
Fruit-Filled	21	33 (5)	20	31	33	34	43	33 (5)	20	31	33	34	43	100%
Other Cookies (e.g., macaroons, biscotti)	116	31 (11)	0	26	30	36	94	31 (11)	0	26	30	36	94	100%
Sandwich Cookies	64	35 (7)	0	32	35	40	53	35 (7)	0	32	35	40	53	100%
Shortbread	8	20 (10)	0	16	22	27	30	20 (10)	0	16	22	27	30	100%
Social Tea/Sugar-Type	53	24 (8)	0	19	23	29	43	24 (8)	0	19	23	29	43	100%
Sugar Wafer	30	35 (17)	0	26	40	47	58	35 (17)	0	26	40	47	58	100%
*Dough and Pastry*	*62*	*6 (8)*	*0*	*0*	*4*	*7*	*29*	*5 (8)*	*0*	*0*	*3*	*5*	*28*	*78%*
Pie Dough and Shells	49	6 (8)	0	0	4	8	29	6 (8)	0	0	2	6	28	79%
Pizza Crust	13	3 (1)	2	3	4	4	6	3 (1)	1	2	3	3	5	77%
*Other Bakery Products*	*416*	*6 (6)*	*0*	*0*	*5*	*8*	*30*	*5 (6)*	*0*	*0*	*4*	*7*	*30*	*85%*
*Pies, Tarts, Cobblers, Crisps*	*100*	*20 (8)*	*2*	*16*	*20*	*26*	*42*	*17 (8)*	*0*	*11*	*16*	*22*	*40*	*75%*
Butter/Sugar	28	27 (5)	18	25	27	29	42	23 (5)	15	21	24	26	40	86%
Custard-Based	13	27 (7)	14	23	26	32	36	23 (7)	9	19	23	29	33	86%
Fruit-Filled Pies	59	16 (6)	2	14	16	18	36	12 (6)	0	10	12	14	33	67%
Beverages ^3^	1407	8 (5)	0	4	9	11	17	7 (5)	0	1	9	11	17	86%
*Dairy and Alternatives*	*242*	*6 (4)*	*0*	*3*	*5*	*10*	*15*	*3 (3)*	*0*	*0*	*3*	*6*	*12*	*49%*
Drinkable Yogurt	35	11 (3)	3	11	12	13	14	7 (3)	0	7	8	10	11	63%
Milk, Flavoured	28	10 (2)	5	9	10	11	12	5 (2)	0	4	6	7	8	52%
Milk, Plain	52	5 (0)	3	4	4	5	6	0 (0)	0	0	0	0	0	0%
Plant-Based Milk, Flavoured	55	5 (2)	0	4	5	7	10	4 (3)	0	3	4	7	9	79%
Plant-Based Milk, Plain	59	2 (1)	0	0	2	3	5	1 (1)	0	0	1	2	5	54%
Shakes	11	12 (4)	6	6	14	14	15	9 (4)	3	3	11	11	12	70%
Smoothies	2	8 (2)	7	7	8	10	10	5 (2)	4	4	5	6	6	60%
*Energy Drinks*	*14*	*7 (5)*	*0*	*0*	*7*	*11*	*14*	*7 (5)*	*0*	*0*	*7*	*11*	*14*	*100%*
Energy Drinks, Diet or Light	7	2 (2)	0	0	0	4	4	2 (2)	0	0	0	4	4	100%
Energy Drinks, Regular	7	12 (1)	11	11	11	13	14	12 (1)	11	11	11	13	14	100%
*Fruit Drinks*	*654*	*10 (3)*	*0*	*9*	*10*	*12*	*17*	*10 (3)*	*0*	*9*	*10*	*12*	*17*	*100%*
Fruit Drink	40	9 (2)	3	10	10	10	12	9 (2)	3	10	10	10	12	100%
Fruit Juice	378	10 (3)	0	9	10	12	17	10 (3)	0	9	10	12	17	100%
Fruit Juice-Drink, Combination	236	10 (3)	0	9	11	12	17	10 (3)	0	9	11	12	17	100%
*Hot Beverages*	*58*	*7 (4)*	*0*	*4*	*9*	*11*	*14*	*6 (4)*	*0*	*3*	*7*	*9*	*13*	*83%*
Cocoa	27	9 (3)	2	8	10	11	14	7 (4)	0	6	9	9	12	70%
Coffee, Flavoured/Sweetened	27	5 (4)	0	1	5	9	13	5 (4)	0	1	5	9	13	100%
Tea, Sweetened	4	6 (4)	1	3	6	9	9	4 (3)	0	2	5	7	7	65%
*Other Beverages*	*39*	*1 (3)*	*0*	*0*	*0*	*0*	*15*	*1 (3)*	*0*	*0*	*0*	*0*	*15*	*67%*
*Soft Drinks*	*272*	*7 (5)*	*0*	*0*	*9*	*11*	*16*	*7 (5)*	*0*	*0*	*9*	*11*	*16*	*100%*
Iced Tea, Diet or Light	13	1 (1)	0	0	0	0	4	1 (1)	0	0	0	0	4	100%
Iced Tea, Regular	52	8 (2)	0	7	9	10	12	8 (2)	0	7	9	10	12	100%
Soft Drink, Regular	121	11 (2)	5	10	11	13	16	11 (2)	5	10	11	13	16	100%
Soft Drink, Diet or Light	86	0 (0)	0	0	0	0	0	0 (0)	0	0	0	0	0	.
*Sports Drinks*	*30*	*4 (2)*	*0*	*2*	*6*	*6*	*6*	*4 (2)*	*0*	*2*	*6*	*6*	*6*	*100%*
Sports Drinks, Diet or Light	11	1 (1)	0	0	2	2	3	1 (1)	0	0	2	2	3	100%
Sports Drinks, Regular	19	6 (0)	5	6	6	6	6	6 (0)	5	6	6	6	6	100%
*Vegetable Drinks*	*43*	*3 (1)*	*1*	*2*	*3*	*4*	*6*	*1 (1)*	*0*	*0*	*0*	*1*	*4*	*16%*
*Water*	*55*	*0 (0)*	*0*	*0*	*0*	*0*	*0*	*0 (0)*	*0*	*0*	*0*	*0*	*0*	*.*
Cereals and Grain Products	969	8 (11)	0	0	3	14	53	6 (10)	0	0	0	10	53	33%
*Hot Breakfast Cereal*	*107*	*12 (13)*	*0*	*0*	*3*	*24*	*42*	*11 (13)*	*0*	*0*	*0*	*23*	*41*	*76%*
Flavoured/Sweetened	48	26 (7)	9	21	25	29	42	25 (8)	0	20	25	29	41	96%
Plain	59	1 (2)	0	0	0	0	10	0 (0)	0	0	0	0	0	0%
*Other Cereals and Grains*	*612*	*2 (2)*	*0*	*0*	*2*	*4*	*29*	*0 (1)*	*0*	*0*	*0*	*0*	*27*	*0%*
*Ready-to-Eat Breakfast Cereal*	*250*	*21 (10)*	*0*	*16*	*20*	*26*	*53*	*17 (10)*	*0*	*11*	*17*	*22*	*53*	*76%*
Flakes	36	18 (10)	7	10	13	23	43	13 (11)	1	5	8	18	40	60%
Flakes w/ Fruit and/or Nuts	30	25 (6)	16	22	24	28	42	21 (6)	11	17	19	23	38	81%
Granola/Muesli	84	21 (5)	4	18	22	25	32	16 (6)	0	13	17	21	28	72%
High-Fibre Compact	8	16 (8)	0	13	18	20	25	16 (8)	0	13	18	20	25	100%
Puffed	13	15 (17)	0	3	10	22	53	15 (17)	0	3	10	22	53	100%
Semi-Compact/Formed	59	25 (12)	3	16	20	34	53	21 (13)	0	11	15	31	51	74%
Shredded	20	16 (8)	0	15	18	22	24	16 (8)	0	15	18	22	24	100%
Dairy Products and Substitutes	1003	6 (9)	0	0	3	10	77	3 (8)	0	0	0	5	76	41%
*Cottage Cheese*	*26*	*5 (2)*	*3*	*4*	*5*	*5*	*12*	*1 (2)*	*0*	*0*	*0*	*0*	*8*	*12%*
Cottage Cheese, Flavoured	5	10 (2)	8	8	10	12	12	6 (2)	4	4	6	8	8	57%
Cottage Cheese, Plain	21	4 (1)	3	4	4	5	6	0 (0)	0	0	0	0	1	2%
*Cream or Cream Substitute*	*85*	*14 (13)*	*0*	*6*	*11*	*22*	*58*	*13 (13)*	*0*	*0*	*10*	*22*	*57*	*73%*
Cream, Aerosol or Whipped	27	16 (5)	7	11	17	22	25	15 (6)	4	11	15	20	23	90%
Cream, Liquid	50	15 (15)	0	6	7	33	58	13 (16)	0	0	3	33	57	61%
Cream, Powder	8	0 (0)	0	0	0	0	0	0 (0)	0	0	0	0	0	.
*Cream Cheese*	*65*	*7 (4)*	*0*	*7*	*7*	*7*	*25*	*1 (4)*	*0*	*0*	*0*	*0*	*20*	*9%*
Cream Cheese, Flavoured	37	8 (4)	0	7	7	7	25	2 (5)	0	0	0	1	20	15%
Cream Cheese, Plain	28	5 (2)	0	3	7	7	10	0 (0)	0	0	0	0	0	0%
*Milk, Condensed*	*36*	*18 (25)*	*0*	*2*	*6*	*37*	*77*	*15 (26)*	*0*	*0*	*0*	*30*	*76*	*29%*
Coconut Milk (canned)	18	7 (18)	0	1	2	2	77	5 (18)	0	0	0	0	76	11%
Condensed Milk	8	58 (2)	57	57	57	61	62	57 (2)	55	55	55	59	60	97%
Evaporated Milk	10	7 (2)	6	6	6	6	13	0 (0)	0	0	0	0	0	0%
*Other Dairy Products and Substitutes*	*468*	*1 (6)*	*0*	*0*	*0*	*0*	*60*	*0 (2)*	*0*	*0*	*0*	*0*	*9*	*42%*
Cheese, unless listed separately	327	0 (3)	0	0	0	0	50	0 (0)	0	0	0	0	0	0%
Dairy-Free Cheese and Spreads	13	1 (1)	0	0	0	0	4	0 (0)	0	0	0	0	0	0%
Feta and Feta-Style Cheese	40	0 (0)	0	0	0	0	0	0 (0)	0	0	0	0	0	.
Hard Cheese	32	7 (18)	0	0	0	0	60	0 (0)	0	0	0	0	0	0%
Processed Cheese	56	7 (3)	0	5	7	10	13	4 (4)	0	0	4	9	9	53%
*Soft Cheese*	*71*	*3 (5)*	*0*	*0*	*2*	*4*	*18*	*2 (4)*	*0*	*0*	*0*	*1*	*17*	*40%*
Soft Cheese, Savoury/Plain	11	4 (1)	3	3	4	4	5	0 (0)	0	0	0	0	0	0%
Soft Cheese, Sweet	45	1 (1)	0	0	0	0	4	0 (0)	0	0	0	0	2	13%
Ricotta	15	11 (4)	3	10	10	11	18	10 (4)	2	9	9	10	17	88%
*Sour Cream*	*17*	*5 (2)*	*3*	*3*	*6*	*6*	*7*	*0 (0)*	*0*	*0*	*0*	*0*	*0*	*0%*
*Yogurt*	*235*	*9 (4)*	*1*	*4*	*11*	*12*	*17*	*6 (4)*	*0*	*0*	*8*	*9*	*14*	*48%*
Yogurt, Flavoured	206	10 (4)	2	7	11	12	17	6 (4)	0	3	8	9	14	55%
Yogurt, Plain	29	3 (1)	1	3	3	4	6	0 (0)	0	0	0	0	0	0%
Desserts ^3,4^	940	19 (19)	0	11	15	20	160	17 (20)	0	8	12	17	160	81%
*Custard, Gelatin, Mousse and Pudding*	*195*	*13 (7)*	*0*	*10*	*15*	*18*	*26*	*12 (7)*	*0*	*9*	*14*	*16*	*26*	*86%*
Custard	8	12 (4)	9	10	11	14	19	12 (4)	8	9	11	14	19	94%
Gelatin	80	12 (8)	0	0	15	15	26	12 (8)	0	0	15	15	26	100%
Mousse	6	5 (5)	0	3	4	10	12	5 (4)	0	2	3	9	11	83%
Pudding	101	15 (6)	0	12	16	19	22	12 (6)	0	11	14	16	21	78%
*Frozen Desserts*	*626*	*14 (5)*	*0*	*11*	*14*	*17*	*31*	*11 (5)*	*0*	*7*	*10*	*14*	*30*	*75%*
Bars	134	19 (5)	0	16	20	23	31	17 (5)	0	14	18	21	29	89%
Cones, Filled	25	16 (4)	3	15	16	19	20	14 (4)	0	12	13	16	17	79%
Dairy-Free	14	11 (2)	10	10	11	13	14	9 (2)	7	7	10	11	12	80%
Frozen Yogurt	56	12 (2)	9	11	12	13	24	9 (2)	6	8	9	10	21	75%
Ice Cream, Ice Milk	292	12 (3)	4	10	12	14	23	8 (3)	0	6	8	10	20	65%
Ice Pops, Juice Bars, Cups	37	17 (6)	0	15	17	20	30	17 (6)	0	15	17	20	30	100%
Sandwiches	34	13 (3)	4	12	13	15	20	9 (3)	0	8	9	11	17	69%
Sherbet and Sorbet	24	16 (4)	10	13	16	19	25	14 (4)	7	11	14	17	25	85%
Sundaes	10	14 (3)	10	12	15	17	18	11 (3)	7	9	12	14	16	79%
*Toppings and Fillings*	*119*	*56 (33)*	*0*	*28*	*53*	*70*	*160*	*56 (33)*	*0*	*28*	*53*	*70*	*160*	*100%*
Cake Frostings and Icings	60	71 (35)	0	46	65	100	160	71 (35)	0	46	65	100	160	100%
Pie Fillings	31	26 (9)	8	21	25	28	53	26 (9)	8	21	25	28	53	100%
Toppings, Dips, Spreads	28	57 (22)	0	50	55	69	113	57 (22)	0	50	55	69	113	100%
Fats, Oils and Vinegars	592	6 (9)	0	0	0	7	63	4 (7)	0	0	0	6	43	81%
*Butter, Margarine, Oils*	*242*	*0 (0)*	*0*	*0*	*0*	*0*	*0*	*0 (0)*	*0*	*0*	*0*	*0*	*0*	*.*
*Mayonnaise*	*39*	*5 (6)*	*0*	*0*	*0*	*13*	*20*	*5 (6)*	*0*	*0*	*0*	*13*	*20*	*100%*
*Salad Dressing*	*311*	*10 (11)*	*0*	*0*	*7*	*19*	*63*	*7 (8)*	*0*	*0*	*5*	*12*	*43*	*79%*
Salad Dressings	253	9 (8)	0	6	7	13	43	8 (8)	0	2	6	13	43	91%
Vinegars	58	16 (17)	0	0	13	31	63	2 (7)	0	0	0	0	42	12%
Fish and Seafood	434	1 (2)	0	0	0	1	22	1 (2)	0	0	0	1	22	85%
Fruits	444	25 (23)	0	9	14	38	83	8 (15)	0	0	0	9	73	30%
*Canned Fruit*	*157*	*12 (4)*	*4*	*10*	*12*	*14*	*31*	*7 (4)*	*0*	*5*	*7*	*10*	*28*	*54%*
Canned in Juice	70	12 (3)	5	9	12	14	16	7 (3)	0	4	7	9	11	53%
Canned in Syrup	73	13 (3)	8	11	13	15	31	9 (3)	4	7	8	11	28	66%
Canned in Water	14	5 (1)	4	4	5	6	8	0 (0)	0	0	0	0	0	0%
*Dried Fruit*	*152*	*51 (20)*	*7*	*33*	*55*	*68*	*83*	*15 (23)*	*0*	*0*	*0*	*32*	*73*	*22%*
Sweetened Dried Fruit	51	65 (14)	33	65	68	76	83	44 (17)	0	32	38	65	73	66%
Unsweetened Dried Fruit	101	43 (18)	7	33	38	60	75	0 (0)	0	0	0	0	0	0%
*Frozen Fruit*	*61*	*8 (3)*	*4*	*6*	*7*	*9*	*15*	*0 (0)*	*0*	*0*	*0*	*0*	*0*	*0%*
*Fruit Sauces*	*62*	*12 (3)*	*7*	*9*	*10*	*15*	*20*	*3 (3)*	*0*	*0*	*0*	*5*	*11*	*16%*
Fruit Sauce, Sweetened	27	15 (2)	13	14	15	16	20	6 (2)	0	5	5	7	11	38%
Fruit Sauce, Unsweetened	35	9 (1)	7	8	9	10	13	0 (0)	0	0	0	0	0	0%
*Other Fruits*	*12*	*14 (21)*	*0*	*0*	*0*	*40*	*50*	*14 (21)*	*0*	*0*	*0*	*40*	*50*	*80%*
Fruit Garnish (e.g., maraschino cherries)	4	43 (5)	40	40	40	45	50	42 (5)	40	40	40	45	50	100%
Fruit Juice Ingredients	8	0 (0)	0	0	0	0	1	0 (0)	0	0	0	0	0	0%
Meat, Eggs and Substitutes	959	2 (3)	0	0	0	2	21	1 (3)	0	0	0	2	21	80%
*Bacon*	*58*	*0 (1)*	*0*	*0*	*0*	*0*	*4*	*0 (1)*	*0*	*0*	*0*	*0*	*4*	*100%*
Cooked	20	0 (1)	0	0	0	0	4	0 (1)	0	0	0	0	4	100%
Uncooked	38	0 (1)	0	0	0	0	2	0 (1)	0	0	0	0	2	100%
*Deli Meats*	*257*	*1 (1)*	*0*	*0*	*0*	*2*	*5*	*1 (1)*	*0*	*0*	*0*	*2*	*5*	*93%*
Dry-cured	90	1 (1)	0	0	0	1	3	1 (1)	0	0	0	0	3	90%
Fully Cooked	167	1 (1)	0	0	1	2	5	1 (1)	0	0	0	2	5	94%
*Eggs and Egg Substitutes*	*56*	*0 (2)*	*0*	*0*	*0*	*0*	*10*	*0 (2)*	*0*	*0*	*0*	*0*	*10*	*100%*
*Meat and Poultry*	*498*	*2 (3)*	*0*	*0*	*1*	*3*	*21*	*2 (3)*	*0*	*0*	*0*	*2*	*21*	*78%*
*Meat Substitutes*	*90*	*2 (2)*	*0*	*0*	*1*	*2*	*11*	*1 (2)*	*0*	*0*	*1*	*2*	*11*	*63%*
Meat Analogues	74	2 (2)	0	0	1	2	11	1 (2)	0	0	1	2	11	66%
Plain Tofu	10	1 (1)	0	0	0	1	2	0 (0)	0	0	0	0	0	0%
Seasoned Tofu and Tempeh	3	4 (4)	0	0	4	9	9	4 (4)	0	0	4	9	9	100%
Sweetened Tofu	3	11 (1)	10	10	11	11	11	10 (1)	10	10	10	11	11	96%
Mixed Dishes, Sides and Entrees	1580	3 (2)	0	1	2	4	20	2 (2)	0	0	1	2	19	48%
*Beans*	*36*	*6 (4)*	*0*	*1*	*7*	*8*	*12*	*5 (4)*	*0*	*0*	*6*	*8*	*12*	*79%*
Baked Beans	26	8 (2)	4	6	8	9	12	7 (2)	4	6	8	8	12	95%
Refried Beans	10	1 (0)	0	0	1	1	1	0 (0)	0	0	0	0	0	17%
*Canned Chili*	*21*	*2 (1)*	*1*	*2*	*2*	*3*	*4*	*1 (1)*	*0*	*0*	*1*	*1*	*2*	*22%*
*Mixed Dishes, Other*	*37*	*3 (2)*	*0*	*1*	*3*	*4*	*8*	*1 (1)*	*0*	*0*	*1*	*2*	*6*	*28%*
Other Mixed Dishes	17	2 (2)	0	1	1	3	8	1 (2)	0	0	0	0	6	17%
Taco Kits	20	3 (1)	2	3	4	4	6	1 (1)	0	1	2	2	4	36%
*Pizza and Frozen Sandwiches*	*214*	*3 (2)*	*1*	*2*	*3*	*4*	*10*	*3 (2)*	*0*	*1*	*2*	*3*	*9*	*72%*
Pizza	161	3 (2)	1	2	3	4	8	3 (2)	0	1	2	4	7	71%
Pizza Snacks and Sandwiches	53	4 (2)	1	3	3	4	10	3 (2)	0	2	2	3	9	76%
*Potatoes*	*126*	*1 (2)*	*0*	*0*	*1*	*2*	*9*	*0 (1)*	*0*	*0*	*0*	*0*	*8*	*20%*
Fries	49	1 (3)	0	0	0	1	8	0 (0)	0	0	0	0	1	14%
Hash Browns and Patties	19	0 (1)	0	0	0	1	2	0 (0)	0	0	0	0	1	42%
Mashed and Scalloped	58	2 (1)	0	1	1	2	9	1 (1)	0	0	0	0	8	19%
*Prepared Salads*	*61*	*5 (4)*	*0*	*2*	*3*	*6*	*16*	*4 (4)*	*0*	*1*	*3*	*6*	*15*	*75%*
Coleslaw	6	14 (1)	12	13	14	15	16	13 (1)	12	12	13	15	15	95%
Fish and Meat Salad	11	3 (3)	0	2	2	3	9	3 (3)	0	2	2	3	9	100%
Grain-Based Salad	6	4 (6)	1	1	2	6	15	3 (6)	0	0	1	5	14	40%
Pasta Salad	8	4 (2)	1	2	5	6	6	3 (2)	0	1	3	5	5	58%
Potato Salad	8	4 (1)	3	3	4	5	6	4 (1)	2	3	3	4	6	82%
Vegetable Salad	22	5 (4)	0	2	3	9	12	4 (4)	0	1	2	8	11	72%
*Refrigerated or Frozen*	*775*	*3 (2)*	*0*	*1*	*2*	*4*	*20*	*2 (2)*	*0*	*0*	*1*	*2*	*19*	*46%*
170–285 g	290	2 (2)	0	1	2	3	12	1 (2)	0	0	1	2	12	43%
Less than 170 g	381	3 (3)	0	1	2	4	20	2 (2)	0	0	1	2	19	45%
More than 285 g	104	3 (2)	0	1	2	4	16	2 (3)	0	0	2	3	15	56%
*Shelf-Stable, Grain-Based Dishes*	*310*	*2 (2)*	*0*	*1*	*2*	*3*	*9*	*1 (1)*	*0*	*0*	*0*	*1*	*8*	*36%*
Pasta and Noodles	177	3 (1)	0	2	3	4	7	1 (1)	0	0	1	1	5	25%
Rice and Grains	116	1 (1)	0	0	1	1	9	1 (1)	0	0	0	1	8	51%
Stuffing	17	2 (1)	0	2	2	3	5	2 (1)	0	1	2	2	4	79%
Nuts and Seeds	205	5 (4)	0	3	4	7	28	1 (4)	0	0	0	0	25	11%
*Butters, Pastes and Creams*	*78*	*8 (6)*	*0*	*6*	*7*	*8*	*28*	*3 (5)*	*0*	*0*	*0*	*3*	*25*	*30%*
Other than Peanut Butter	28	4 (4)	0	0	3	7	13	2 (4)	0	0	0	2	10	28%
Peanut Butter	50	9 (5)	6	7	7	13	28	4 (6)	0	0	2	9	25	31%
*Nuts and Seeds*	*127*	*4 (2)*	*0*	*2*	*3*	*4*	*13*	*0 (0)*	*0*	*0*	*0*	*0*	*0*	*0%*
Nut and Seed Flours	7	7 (5)	0	4	7	13	13	0 (0)	0	0	0	0	0	0%
Nuts and Seeds, Not for Snacking	120	3 (2)	0	2	3	4	8	0 (0)	0	0	0	0	0	0%
Other Foods and Beverages	274	6 (13)	0	0	0	8	100	6 (13)	0	0	0	6	100	87%
Baking Misc. (e.g., yeast, baking soda)	*15*	*0 (0)*	*0*	*0*	*0*	*0*	*0*	*0 (0)*	*0*	*0*	*0*	*0*	*0*	*.*
Seasoning, Topping, Breading Mix	*259*	*8 (14)*	*0*	*0*	*0*	*13*	*100*	*7 (14)*	*0*	*0*	*0*	*9*	*100*	*86%*
Sauces, Dips and Condiments	1204	11 (14)	0	2	5	17	70	10 (15)	0	0	2	17	70	63%
*Condiments*	*291*	*18 (17)*	*0*	*0*	*19*	*33*	*66*	*18 (17)*	*0*	*0*	*17*	*31*	*66*	*96%*
Barbecue and Steak Sauce	115	30 (14)	0	21	31	38	66	30 (14)	0	21	31	38	66	100%
Ketchup	24	24 (8)	7	20	27	27	33	18 (8)	0	14	21	21	29	70%
Mustard	54	9 (15)	0	0	0	20	60	9 (15)	0	0	0	20	60	100%
Other Condiments (e.g., hot sauce)	98	9 (12)	0	0	3	17	60	9 (12)	0	0	3	17	60	97%
*Dips*	*259*	*3 (3)*	*0*	*0*	*3*	*4*	*25*	*1 (3)*	*0*	*0*	*0*	*1*	*23*	*24%*
Dips and Salsa	210	4 (3)	0	3	3	5	25	1 (3)	0	0	0	1	23	23%
Hummus and Legume Dips	49	1 (2)	0	0	0	3	10	1 (2)	0	0	0	0	9	29%
*Sauces*	*654*	*11 (14)*	*0*	*2*	*5*	*13*	*70*	*10 (15)*	*0*	*0*	*3*	*13*	*70*	*65%*
Curry Paste	27	6 (4)	0	3	5	7	16	3 (4)	0	0	0	5	14	37%
Gravy and Cooking Sauce	188	8 (12)	0	0	3	9	48	8 (12)	0	0	3	8	48	79%
Marinades	60	15 (14)	0	5	11	22	50	15 (14)	0	5	11	22	50	100%
Pesto	15	3 (6)	0	0	0	5	20	0 (1)	0	0	0	0	2	29%
Soya and Oriental Sauce	61	16 (17)	0	7	12	24	70	16 (17)	0	7	12	24	70	98%
Sweet Sauce (e.g., plum sauce)	73	35 (14)	0	27	34	43	63	35 (14)	0	27	34	43	63	100%
Tomato Sauce	198	4 (2)	0	3	5	5	8	2 (2)	0	0	2	3	6	37%
White Sauce	32	3 (2)	2	2	3	4	7	1 (2)	0	0	1	1	5	26%
Snacks	854	10 (16)	0	2	4	8	83	5 (10)	0	0	0	4	65	44%
*Chips, Corn and Rice Snacks*	*412*	*4 (5)*	*0*	*0*	*3*	*5*	*35*	*3 (5)*	*0*	*0*	*1*	*3*	*34*	*59%*
Extruded Snacks (e.g., cheese puffs)	90	6 (8)	0	2	5	7	35	5 (8)	0	0	1	6	34	52%
Flavoured Chips	194	4 (3)	0	2	4	4	15	3 (3)	0	1	3	3	14	68%
Plain Chips	116	2 (4)	0	0	0	2	30	1 (2)	0	0	0	0	17	32%
Savoury Snack Mixes	12	5 (2)	2	3	5	6	9	3 (2)	1	2	4	5	8	65%
*Ethnic Snacks*	*21*	*7 (12)*	*0*	*2*	*4*	*8*	*54*	*5 (12)*	*0*	*0*	*0*	*6*	*53*	*38%*
*Fruit Snacks (e.g., apple chips, fruit leather)*	*40*	*61 (24)*	*6*	*49*	*72*	*78*	*83*	*9 (14)*	*0*	*0*	*0*	*24*	*37*	*12%*
*Meat Snacks*	*42*	*9 (11)*	*0*	*0*	*4*	*18*	*43*	*9 (11)*	*0*	*0*	*4*	*18*	*43*	*96%*
Meat and Poultry Jerky	20	19 (9)	7	12	18	21	43	19 (9)	7	12	18	21	43	100%
Meat and Poultry Sticks	22	1 (1)	0	0	0	2	4	1 (1)	0	0	0	2	4	88%
*Nuts and Seeds*	*225*	*13 (13)*	*0*	*4*	*6*	*22*	*52*	*4 (9)*	*0*	*0*	*0*	*3*	*41*	*15%*
Mix w/ Fruit, Chocolate, Candy	80	28 (10)	10	20	28	33	52	12 (12)	0	0	11	17	41	34%
Mix w/o Fruit, Chocolate, Candy	145	5 (2)	0	4	4	6	14	0 (1)	0	0	0	0	8	5%
*Popcorn*	*80*	*9 (17)*	*0*	*0*	*0*	*6*	*65*	*9 (17)*	*0*	*0*	*0*	*4*	*65*	*63%*
Plain/Savoury	61	1 (2)	0	0	0	2	6	0 (1)	0	0	0	0	6	30%
Sweet	19	35 (17)	0	24	34	42	65	35 (17)	0	24	34	42	65	100%
*Pretzels*	*34*	*12 (15)*	*0*	*2*	*4*	*14*	*43*	*11 (15)*	*0*	*1*	*3*	*13*	*42*	*80%*
Coated or Filled	14	26 (14)	8	14	23	43	43	25 (14)	7	13	22	42	42	97%
Plain	20	2 (2)	0	2	2	4	5	2 (1)	0	0	1	3	4	66%
Soups	464	1 (1)	0	0	1	2	7	1 (1)	0	0	0	1	7	52%
*Bouillon and Broth*	*110*	*0 (0)*	*0*	*0*	*0*	*0*	*1*	*0 (0)*	*0*	*0*	*0*	*0*	*1*	*94%*
Broth	56	0 (0)	0	0	0	0	1	0 (0)	0	0	0	0	1	90%
Dry Mix	39	0 (0)	0	0	0	0	1	0 (0)	0	0	0	0	1	100%
Liquid Concentrates	15	0 (0)	0	0	0	0	0	0 (0)	0	0	0	0	0	100%
*Canned Condensed Soup*	*76*	*1 (1)*	*0*	*0*	*1*	*2*	*6*	*1 (1)*	*0*	*0*	*0*	*1*	*6*	*45%*
Cream or Cheese	32	1 (1)	0	0	1	2	2	0 (0)	0	0	0	0	1	12%
Non-Cream	44	2 (2)	0	0	1	2	6	1 (2)	0	0	1	2	6	69%
*Dry Soup Mix*	*55*	*1 (1)*	*0*	*0*	*1*	*1*	*4*	*0 (1)*	*0*	*0*	*0*	*1*	*3*	*37%*
Cream or Cheese	15	2 (1)	0	1	2	3	3	1 (1)	0	1	1	1	2	53%
Non-Cream	40	1 (1)	0	0	0	1	4	0 (1)	0	0	0	0	3	30%
*Fresh and Instant Oriental Noodle*	*76*	*1 (1)*	*0*	*0*	*1*	*1*	*3*	*1 (1)*	*0*	*0*	*1*	*1*	*3*	*87%*
*Ready-to-Serve Soup*	*147*	*2 (1)*	*0*	*1*	*2*	*3*	*7*	*1 (1)*	*0*	*0*	*0*	*2*	*7*	*30%*
Cream or Cheese	28	2 (2)	0	0	1	4	7	2 (2)	0	0	1	3	7	65%
Non-Cream Soup	119	2 (1)	0	1	2	3	6	1 (1)	0	0	0	1	5	23%
Sugars and Sweets	776	51 (17)	0	43	50	60	100	51 (17)	0	43	50	60	100	100%
*Confectionery*	*469*	*51 (16)*	*0*	*45*	*51*	*60*	*100*	*51 (16)*	*0*	*45*	*51*	*60*	*100*	*100%*
Baking Candies (e.g., sprinkles, chocolate chips)	33	53 (19)	0	47	53	62	100	53 (19)	0	47	53	62	100	100%
Breath Mints	6	94 (5)	88	93	93	100	100	94 (5)	88	93	93	100	100	100%
Candies (e.g., licorice, gummies, jelly beans)	151	56 (12)	6	48	55	63	100	56 (12)	6	48	55	63	100	100%
Chocolate and Candy Bars	257	46 (15)	0	42	49	54	80	46 (15)	0	42	49	54	80	100%
Hard Candies	9	70 (17)	48	61	68	80	94	70 (17)	48	61	68	80	94	100%
Marshmallows	13	56 (4)	52	52	54	57	67	56 (4)	52	52	54	57	67	100%
*Sugar*	*7*	*98 (4)*	*89*	*100*	*100*	*100*	*100*	*98 (4)*	*89*	*100*	*100*	*100*	*100*	*100%*
Icing Sugar	1	89 (0)	89	89	89	89	89	89 (0)	89	89	89	89	89	100%
Sugar	6	100 (0)	100	100	100	100	100	100 (0)	100	100	100	100	100	100%
*Sweet Condiments*	*300*	*50 (17)*	*0*	*40*	*50*	*60*	*81*	*50 (17)*	*0*	*40*	*50*	*60*	*81*	*100%*
Bread Spreads (e.g., chocolate spread)	13	52 (15)	25	47	50	58	79	52 (15)	25	47	50	58	79	100%
Fruit Preserve Spreads (e.g., jam, jelly)	187	45 (14)	0	35	45	55	75	45 (14)	0	35	45	55	75	100%
Honey and Molasses	38	76 (9)	45	76	80	80	80	76 (9)	45	76	80	80	80	100%
Syrups	62	49 (16)	3	40	50	61	81	49 (16)	3	40	50	61	81	100%
Vegetables	957	3 (6)	0	0	2	4	40	2 (5)	0	0	0	0	40	16%
*Canned Vegetables and Legumes*	*460*	*2 (2)*	*0*	*0*	*1*	*3*	*29*	*0 (1)*	*0*	*0*	*0*	*0*	*6*	*15%*
Canned Tomatoes	93	3 (1)	1	2	3	4	6	0 (1)	0	0	0	0	3	9%
Other Canned Vegetables	367	2 (2)	0	0	1	2	29	0 (1)	0	0	0	0	6	17%
*Dried Legumes*	*86*	*3 (2)*	*0*	*1*	*2*	*3*	*11*	*0 (0)*	*0*	*0*	*0*	*0*	*0*	*0%*
*Fresh Vegetables*	*54*	*3 (6)*	*0*	*0*	*2*	*3*	*40*	*0 (2)*	*0*	*0*	*0*	*0*	*18*	*3%*
*Frozen Vegetables*	*155*	*3 (2)*	*0*	*2*	*2*	*4*	*14*	*0 (0)*	*0*	*0*	*0*	*0*	*4*	*2%*
Frozen Vegetables w/ Sauce	9	2 (2)	0	2	2	3	5	1 (1)	0	0	1	1	4	40%
Frozen Vegetables w/o Sauce	146	3 (2)	0	2	2	4	14	0 (0)	0	0	0	0	0	0%
*Vegetable Paste*	*20*	*6 (5)*	*0*	*0*	*9*	*9*	*13*	*0 (0)*	*0*	*0*	*0*	*0*	*0*	*0%*
Tomato Paste	12	10 (1)	9	9	9	9	13	0 (0)	0	0	0	0	0	0%
Vegetable and Herb Paste	8	0 (0)	0	0	0	0	0	0 (0)	0	0	0	0	0	.
*Pickled Vegetables*	*182*	*8 (10)*	*0*	*0*	*3*	*13*	*40*	*7 (10)*	*0*	*0*	*0*	*13*	*40*	*57%*
Sour or Spicy	126	2 (4)	0	0	0	3	29	0 (1)	0	0	0	0	9	11%
Sweet	56	21 (7)	0	14	20	27	40	21 (7)	0	14	20	27	40	100%
TOTAL	15259	11 (16)	0	1	4	13	160	9 (16)	0	0	1	11	160	62%

^1^ All values presented represent products in their “as consumed” form, prepared according to package directions; ^2^ Free sugar as a percent of total sugar was calculated for each product (*n* = 15,259) and the average of those results is presented here; ^3^ Total and free sugar contents for beverages and desserts presented as g per 100 mL; ^4^ Maximum total sugar content exceeds 100 g per 100 mL due to rounding of total sugar declaration on products with small serving sizes in desserts food group. Abbreviations: NFt = Nutrition Facts table; $\bar{X}$ = mean; SD = standard deviation; w/ = with; w/o = without.
